# Perforin-2 is dispensable for host defense against *Aspergillus fumigatus* and *Candida albicans*

**DOI:** 10.1128/msphere.00803-24

**Published:** 2024-12-20

**Authors:** Mariano A. Aufiero, Li-Yin Hung, De'Broski R. Herbert, Tobias M. Hohl

**Affiliations:** 1 Louis V. Gerstner Jr. Graduate School of Biomedical Sciences, Sloan Kettering Institute, Memorial Sloan Kettering Cancer Center, New York, New York, USA; 2 Department of Pathobiology, School of Veterinary Medicine, University of Pennsylvania, Philadelphia, Pennsylvania, USA; 3 Infectious Disease Service, Memorial Sloan Kettering Cancer Center, New York, New York, USA; 4 Human Oncology and Pathogenesis Program, Memorial Sloan Kettering Cancer Center, New York, New York, USA; University of Georgia, Athens, Georgia, USA

**Keywords:** *Aspergillus fumigatus*, innate immunity, *Candida albicans*, Perforin, neutrophil, fungi, *Mpeg1*

## Abstract

Myeloid phagocytes are essential for antifungal immunity against pulmonary *Aspergillus fumigatus* and systemic *Candida albicans* infections. However, the molecular mechanisms underlying fungal clearance by phagocytes remain incompletely understood. In this study, we investigated the role of Perforin-2 (*Mpeg1*) in antifungal immunity. We found that *Mpeg1*^−/−^ mice generated on a mixed C57BL/6J-DBA/2 background exhibited enhanced survival, reduced lung fungal burden, and greater neutrophil fungal killing activity compared to wild-type C57BL/6J (B6) mice, suggesting that Perforin-2 may impair antifungal immune responses. However, when we compared *Mpeg1*^−/−^ mice with co-housed *Mpeg*^+/+^ littermate controls, these differences were no longer observed, indicating that initial findings were likely influenced by differences in the murine genetic background or the microbiota composition. Furthermore, Perforin-2 was dispensable for antifungal immunity during *C. albicans* bloodstream infection. These results suggest that Perforin-2 is not essential for host defense against fungal infections in otherwise immune-competent mice.

**IMPORTANCE:**

Humans encounter fungal pathogens daily and rely on innate immune cells to clear *Aspergillus fumigatus*, the leading cause of mold pneumonia worldwide, and *Candida albicans*, the most common cause of fungal bloodstream infections. The World Health Organization has classified *A. fumigatus* and *C. albicans* as critical priority fungal pathogens due to the emergence of drug resistance and the increasing number of susceptible individuals across the globe. The mechanisms by which innate immune cells clear these fungal pathogens remain incompletely defined. In this study, we examined the role of a pore-forming protein called Perforin-2 in host defense against these fungal pathogens, in part because Perforin-2 has been implicated in antibacterial host defense. Our findings reveal that Perforin-2 is dispensable for antifungal immunity against respiratory *A. fumigatus* and systemic *C. albicans* infections in mice, suggesting that the antimicrobial activity of Perforin-2 does not extend to these two fungal pathogens.

## OBSERVATION

Myeloid phagocytes are essential for immunity to *Aspergillus fumigatus* and *Candida albicans* (1–5). NADPH oxidase represents a central antifungal effector system against these fungal pathogens, though NADPH oxidase-deficient neutrophils exhibit only a partial *A. fumigatus* conidial killing defect (6). Humans with chronic granulomatous disease, characterized by loss of NADPH oxidase activity, have a 40–55% lifetime risk of infection with *A. fumigatus* despite universal exposure, suggesting the existence of NADPH oxidase-independent effectors (7). Perforin-2 (*Mpeg1*) is a pore-forming protein that is highly expressed by myeloid cells (8, 9). Perforin-2 facilitates dendritic cell IL-33 release during helminth infection (10) and cross-presentation of exogenous antigens (11). Some individuals with nontuberculous mycobacterium infections harbor *Mpeg1* mutations, and introduction of these mutations into the monocyte cell line, THP-1, impaired *Mycobacterium smegmatis*, *Salmonella typhimurium,* and *Staphylococcus aureus* killing (12), implicating Perforin-2 in antibacterial immunity. *In vitro*, *Mpeg1^−/−^* neutrophils and bone marrow-derived macrophages (BMDMs) exhibited defective *Mycobacterium avium* killing (8). Consistent with these findings, *Mpeg1^−/−^* mice were susceptible to cutaneous *S. aureus* and oral *S. typhimurium* infection (8). However, a second study with an independent *Mpeg1^−/−^* strain found that *Mpeg1* was dispensable for bacterial clearance following intranasal infection with *M. tuberculosis*, *S. aureus*, or *L. longbeachae*, and that BMDMs from *Mpeg1^−/−^* had no defect in killing *M. smegmatis, L. pneumophila,* or *S. aureus in vitro* (9). While the role of Perforin-2 in antibacterial immunity remains incompletely defined, its role in antifungal immunity is unknown.

**Fig 1:**
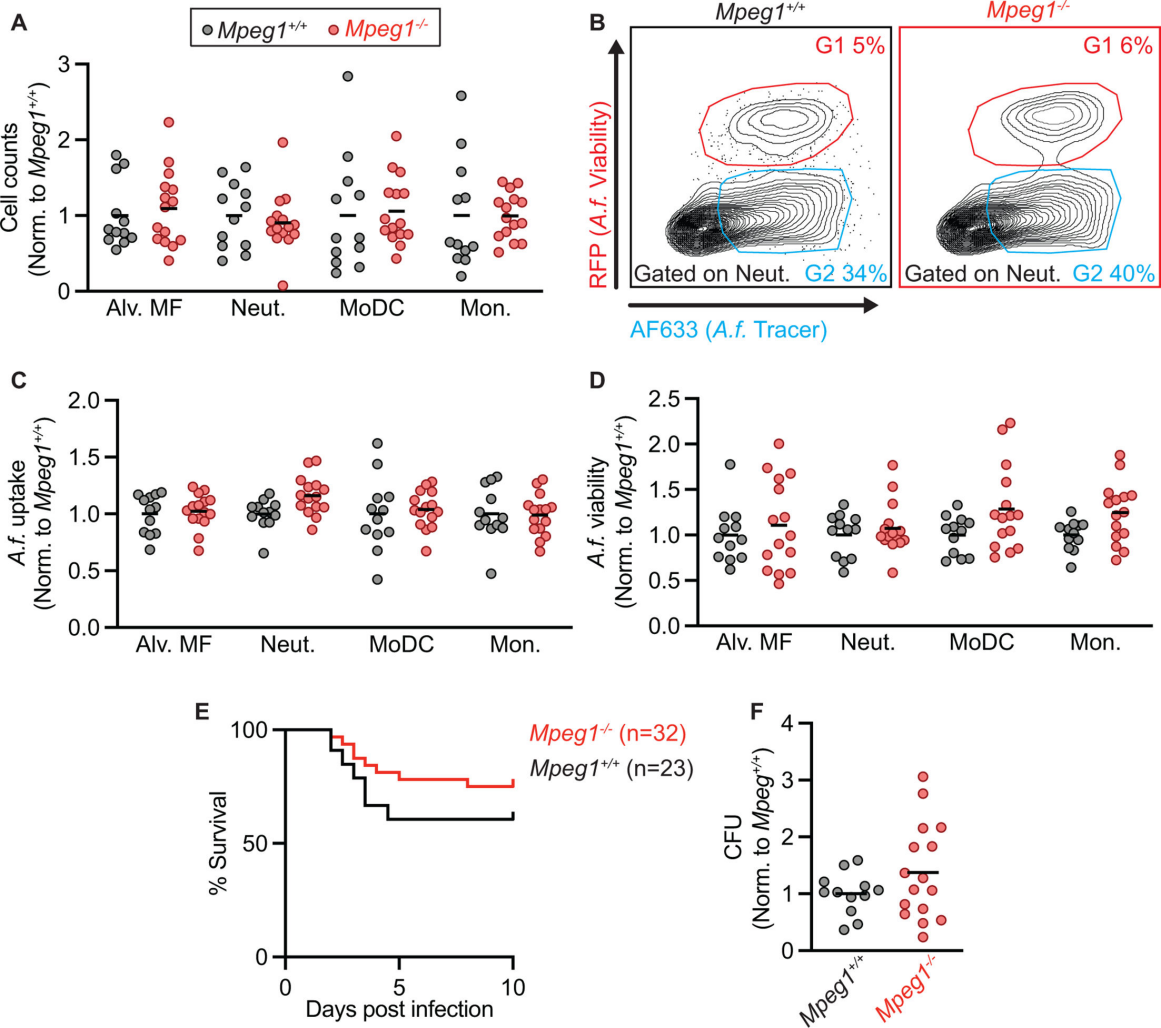
Phenotype of *Mpeg1^−/−^* and littermate control mice following pulmonary *A. fumigatus* infection. (**A**) Alveolar macrophage, neutrophil, Mo-DC, and monocyte numbers in the lungs of *Mpeg1^+/+^* and *Mpeg1^−/−^* mice 24 hpi with 3 × 10^7^ FLARE conidia. (**B**) Representative flow plots of CD45^+^CD11b^+^Ly6G^+^ lung neutrophils from *Mpeg1^+/+^* and *Mpeg1^−/−^* mice analyzed for RFP (*Af* viability fluorophore) and AF633 (*Af* tracer fluorophore) fluorescence. Gate 1 (G1) denotes neutrophils with live conidia, G2 neutrophils with killed conidia. (**C**) Uptake of conidia by and (**D**) conidial viability in indicated lung leukocyte subsets, quantified using FLARE conidia and flow cytometry from infected *Mpeg1^+/+^* and *Mpeg1^−/−^* mice 24 hpi. Fungal uptake by leukocytes = G1 + G2; fungal viability in leukocytes = G1/[G1 + G2]. (**E**) Survival of *Mpeg1^+/+^* and *Mpeg1^−/−^* mice after infection with 4–6 × 10^7^ *A. fumigatus* conidia (*P* = 0.186). Significance calculated by log-rank (Mantel–Cox) test. (**F**) CFU from lungs of *Mpeg1^+/+^* and *Mpeg1^−/−^* mice at 24 hpi with 3 × 10^7^ conidia. (**A, C, D, and E**) Each dot represents a mouse, and the bar indicates mean. Significance calculated by two-way ANOVA with Šídák’s multiple comparison test. Data are pooled from two experiments.

To test whether Perforin-2 contributes to antifungal immunity, we infected *Mpeg1^−/−^* mice on a mixed C57BL/6J-DBA/2 background (10) or C57BL/6J (B6) mice (as controls) with 4–6 × 10^7^ *A. fumigatus* CEA10 conidia intratracheally and monitored survival and quantified lung fungal burden at 24 hours postinfection (hpi). While 60% of B6 mice succumbed to infection, 100% of *Mpeg1^−/−^* mice survived (Fig. S1A). Consistent with these data, *Mpeg1^−/−^* mice had reduced lung fungal burden at 24 hpi (Fig. S1B). To measure the myeloid phagocyte response in the lung, we infected mice with Fluorescent *Aspergillus* reporter (FLARE) conidia that enable us to quantify fungal uptake and killing by myeloid phagocytes (6). Only monocyte-derived dendritic cells (Mo-DCs) were increased in number in *Mpeg1^−/−^* mice compared with B6 controls at 24 hpi (Fig. S2A). Mo-DCs from *Mpeg1^−/−^* mice had greater fungal uptake compared with B6 control mice while inflammatory monocytes had a modest decrease in conidial uptake (Fig. S2B and C). All lung phagocytes examined in *Mpeg1^−/−^* mice had decreased conidial viability compared with B6 mice (Fig. S2B and D), supporting the idea that Perforin-2 impairs the antifungal activity of phagocytes. These results were unexpected since Perforin-2 exhibits antibacterial activity against bacterial pathogens (8). To test the possibility that differences in mouse strain backgrounds or microbiota between *Mpeg1^−/−^* and B6 control mice caused these results, we crossed *Mpeg1^−/−^* mice to C57BL/6J mice and then crossed their progeny to generate *Mpeg1^−/−^* and *Mpeg1^+/+^* littermate controls. At 24 hpi with *A. fumigatus*, there was no difference in lung myeloid phagocyte numbers among *Mpeg1^−/−^* mice and *Mpeg1^+/+^* mice (Fig. 1A) and no difference in the uptake by or in the viability of *A. fumigatus* conidia in any lung phagocyte subset between *Mpeg1^−/−^* mice and *Mpeg1^+/+^* mice (Fig. 1B through D). Moreover, we observed no difference in murine survival (Fig. 1E) and no difference in lung fungal burden by enumerating lung colony-forming units (CFUs) (Fig. 1F) in *Mpeg1^−/−^* mice and *Mpeg1^+/+^* mice. These findings indicate that Perforin-2 is dispensable for phagocyte function during pulmonary *A. fumigatus* infection and does not contribute to lung fungal clearance. Thus, our initial finding that *Mpeg1^−/−^* mice were more resistant than B6 mice to respiratory *A. fumigatus* challenge was due to the differences in the strain background or in the microbiota of these strains, and not to Perforin-2 function.

**Fig 2:**
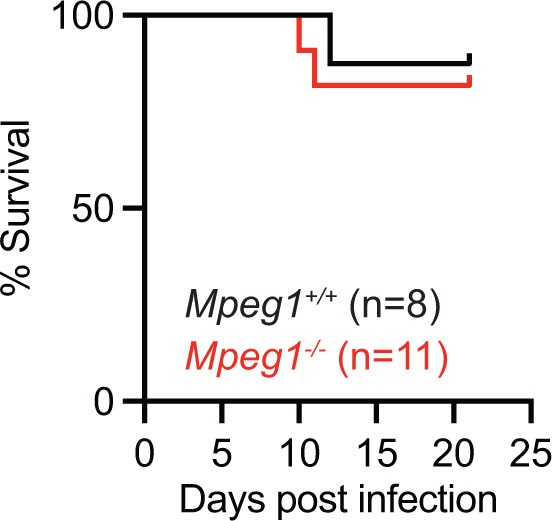
Phenotype of *Mpeg1^−/−^* and littermate control mice following systemic *C. albicans* infection. Survival of *Mpeg1^−/−^* and *Mpeg1^+/+^* mice after intravenous infection with 1.5 × 10^5^ *C. albicans* yeast cells (*P* = 0.696). Significance calculated by log-rank (Mantel–Cox) test. Data are pooled from two experiments.

Finally, we tested whether Perforin-2 is required during systemic candidiasis, the most common type of fungal bloodstream infection (13). We infected mice intravenously with 1.5 × 10^5^ *C. albicans* blastoconidia and monitored for survival. There was no difference in the survival of *Mpeg1^−/−^* mice compared with littermate *Mpeg1^+/+^* controls (Fig. 2). These results indicate that Perforin-2 is dispensable for host defense against systemic *C. albicans* infection as well as *A. fumigatus* lung infection.

While Perforin-2 is present in bacteria-containing phagosomes (8, 9) and associated with the formation of pores on bacterial membranes (8), there are conflicting studies on the susceptibility of *Mpeg1^−/−^* mice to bacterial infection and on the contribution of Perforin-2 to the bactericidal capacity of phagocytes (8, 9). These contradictory findings on the role of Perforin-2 in antibacterial immunity may stem from the differences in infection routes and organ-specific immune responses. Our study and Ebrahimnezhaddarzi et al. utilized models of pulmonary infection with *A. fumigatus* and *M. tuberculosis* or *S. aureus,* respectively, and found no defect in pathogen clearance from the lungs of *Mpeg1^−/−^* mice (9). In contrast, McCormack et al. observed a significant effect of *Mpeg1* deletion on murine survival following orogastric and epicutaneous bacterial infections (8). Differences in inbred mouse strains may contribute to different outcomes since McCormack et al. used 129 × 1/SvJ and mixed C57BL/6J-129X1/SvJ backgrounds (8), while Ebrahimnezhaddarzi et al. used a mixed C57BL6/J-BALB/c background to generate *Mpeg1^−/−^* mice (9). Despite these differences, both studies used littermate controls, minimizing strain-related variations between experimental and control groups. Indeed, our study used *Mpeg1^−/−^* mice generated on a mixed C57BL/6J-DBA/2 background and found significant differences in *Mpeg1^−/−^* mice compared with B6 controls that were lost when comparing with littermates.

Because Perforin-2 likely inserts into target cell membranes to exert its antimicrobial effect, the susceptibility of pathogens to Perforin-2 may be influenced by their cell wall composition, which resides outside the cell membrane in fungi and gram-positive bacteria. The cell wall of gram-positive bacteria like *S. aureus* are primarily composed of peptidoglycan, a polymer of alternating N-acetylglucosamine and N-acetylmuramic acid residues, with peptide side chains that cross-link adjacent glycan chains (14). In contrast, the *A. fumigatus* conidial cell wall consists of a core layer of β-1,3-linked glucan polysaccharides, mannoproteins, galactomannan, and chitin, which are further covered by a layer of hydrophobins and melanin under resting conditions (15). These differences in the cell wall may affect the accessibility of Perforin-2 to the underlying membrane in target cells, which could explain variations in susceptibility to Perforin-2-mediated killing.

These findings expand our understanding of the role of Perforin-2 in antimicrobial immunity and of which factors are essential or redundant for phagocyte killing of *A. fumigatus* conidia.

## Data Availability

Requests for further information, resources, or reagents should be directed to the corresponding author.

## SUPPLEMENTAL MATERIAL

Supplemental Material msphere.00803-24-s0001.docx Description of key reagents and methods used and supplemental figures.

ASM does not own the copyrights to Supplemental Material that may be linked to, or accessed through, an article. The authors have granted ASM a non-exclusive, world-wide license to publish the Supplemental Material files. Please contact the corresponding author directly for reuse.
