# Supplementary material for: Perforin-2 is dispensable for host defense against Aspergillus fumigatus and Candida albicans
Source: mSphere. 2024 Dec 20;10(1):e00803-24. doi: 10.1128/msphere.00803-24 (PMC11774031; doi:10.1128/msphere.00803-24)
Supplement: Supplemental Material — Description of key reagents and methods used and supplemental figures. [file msphere.00803-24-s0001.docx]

**SUPPLEMENTAL METHODS**

*Mice*

C57BL/6J mice (stock # 000664) were purchased from The Jackson Laboratory. *Mpeg1^-/-^* have been previously described (1). In brief, *Mpeg1*-deficient mice were created using CRISPR-Cas9 gene editing at the Penn Vet transgenic core facility. Embryos were collected from 6- to 8-week-old superovulated C57BL/6 females, which had been mated with B6D2F1 males (offspring of C57BL/6 females and DBA/2 males). The CRISPR components were microinjected into the cytoplasm of the embryos, which were then transferred to the oviducts of pseudopregnant Swiss Webster female recipients. Mice that were homozygous for the targeted deletion were outcrossed to C57BL/6J mice, and heterozygous offspring were interbred to establish multiple founder lines. The data presented here were derived from a single founder line. All mice used in this study were 8-12 weeks old. Within experiments, mice were age- and sex-matched. Experiments were performed with both male and female mice. Mice were bred and housed in the Research Animal Resource Center at MSKCC in individual ventilated cages under specific pathogen free conditions. C57BL/6J control mice were housed separately from *Mpeg1^-/-^* mice, but *Mpeg1^+/+^* littermates were co-housed with *Mpeg1^-/-^* mice. Animal experiments were conducted with approval of the MSKCC (protocol 13-07-008) Institutional Animal Care and Use Committee. Animal studies complied with all applicable provisions established by the Animal Welfare Act and the Public Health Services Policy on the Humane Care and Use of Laboratory Animals.

Aspergillus fumigatus *strains and murine infection model*

*A. fumigatus* strains CEA10 and CEA10-RFP (2) were cultured on glucose minimal medium slants at 37°C for 4–7 days prior to harvesting conidia for experimental use. Generation of FLARE conidia has been previously described (3). For infections, mice were lightly anesthetized by isoflurane inhalation and 3-6x10^7^ A. fumigatus conidia were instilled via the intratracheal route in 50 μL of PBS + 0.025% Tween-20.

*Quantification of fungal burden*.

To measure colony-forming units (CFU) in the lungs of infected mice, lungs were dissected and homogenized with a PowerGen 125 homogenizer (Fisher Scientific) for 10-15 seconds in 2 mL of PBS. 10 μL was removed and diluted for plating onto Sabourand dextrose agar plates. Plates were incubated for 48 hours at 37°C and CFU were enumerated by counting.

*Flow cytometry*

For analysis of immune cells, single cell suspensions of mouse lungs were generated and stained for flow cytometry as previously described (4). In brief, dead cells were excluded with DAPI or LiveDead Aqua (ThermoFisher). Neutrophils were identified as CD45+ CD11b+ Ly6G+ Siglec-F- cells, inflammatory monocytes as CD45+ CD11b+ CD11c− Ly6G− Ly6Chi cells, Mo-DCs as CD45+ CD11b+ CD11c+ Ly6G− Ly6Chi MHC class II+ cells, and alveolar macrophages as CD11c+, Siglec-F+. Phagocytes that contain live conidia are RFP+ and AF633+ (G1) and phagocytes that contain dead conidia are RFP- AF633+ (G2). Conidial phagocytosis was quantified as the sum of the fraction of a given phagocyte in the G1 gate and the fraction of a given phagocyte in the G2 gate (G1+G2). To assess how effective phagocytes were at killing conidia, the fraction of viable conidia was calculated as G1/(G1+G2).

*Murine systemic candidiasis infection model*

*C. albicans* strain SC5314 was used in this study and was cultured as previously described (5). Yeast cells were serially passaged 2 times in YPD (yeast extract, bacto-peptone and dextrose) broth, grown at 30˚C with shaking for 18-24 hours at each passage. 1.5x10^5^ yeast cells were injected intravenously via the lateral tail vein.

**SUPPLEMENTAL FIGURES**

|  |
| --- |
| Figure S1. Phenotype of *Mpeg1^-/-^* and C57BL/6J control mice following pulmonary *A. fumigatus* infection*.*  (A) Survival of B6 and *Mpeg1^-/-^* mice after infection with 4-6x10^7^ *A. fumigatus* conidia. Significance calculated by log-rank (Mantel-Cox) test.  (B) CFU from lungs of B6 and *Mpeg1^-/-^* mice at 24 hpi with 3x10^7^ conidia. Each dot represents a mouse, and the bar indicates mean. Significance calculated by Mann-Whitney test. All data are pooled from 2 experiments. |

|  |
| --- |
| *Figure S2.* Mpeg1^-/-^ *phagocytes have superior antifungal activity relative to B6 mice.*  (A) Cell counts in the lungs of B6 and *Mpeg1^-/-^* mice of alveolar macrophages (Alv. MF), neutrophils (Neut.), MoDCs, and monocytes (Mon.) 24 hpi with 3x10^7^ FLARE conidia.  (B) Representative flow plots of CD45^+^CD11b^+^Ly6G^+^ lung neutrophils from *Mpeg1^-/-^* and B6 mice analyzed for RFP (*Af* viability fluorophore) and AF633 (*Af* tracer fluorophore) fluorescence. Gate 1 (G1) denotes neutrophils with live conidia, G2 neutrophils with killed conidia.  (C) Uptake of conidia by and (D) conidial viability in lung neutrophils, quantified using FLARE conidia and flow cytometry from infected B6 and *Mpeg1^-/-^* mice 24 hpi.  (A, C, D) Each dot represents a mouse, and the bar indicates mean. Significance calculated by two-way ANOVA with Šídák’s multiple comparison test. Data are pooled from 2 experiments. |

**SUPPLEMENTAL REFERENCES**

1. Hung L-Y, Tanaka Y, Herbine K, Pastore C, Singh B, Ferguson A, Vora N, Douglas B, Zullo K, Behrens EM, Li Hui Tan T, Kohanski MA, Bryce P, Lin C, Kambayashi T, Reed DR, Brown BL, Cohen NA, Herbert DR. 2020. Cellular context of IL-33 expression dictates impact on anti-helminth immunity. Sci Immunol 5:eabc6259.

2. Liu K-W, Grau MS, Jones JT, Wang X, Vesely EM, James MR, Gutierrez-Perez C, Cramer RA, Obar JJ. 2022. Postinfluenza Environment Reduces Aspergillus fumigatus Conidium Clearance and Facilitates Invasive Aspergillosis *In Vivo*. mBio e02854-22.

3. Jhingran A, Mar KB, Kumasaka DK, Knoblaugh SE, Ngo LY, Segal BH, Iwakura Y, Lowell CA, Hamerman JA, Lin X, Hohl TM. 2012. Tracing conidial fate and measuring host cell antifungal activity using a reporter of microbial viability in the lung. Cell Rep 2:1762–1773.

4. Aufiero MA, Shlezinger N, Gjonbalaj M, Mills KAM, Ballabio A, Hohl TM. 2023. Dectin-1/CARD9 induction of the TFEB and TFE3 gene network is dispensable for phagocyte anti- *Aspergillus* activity in the lung. Infect Immun e00217-23.

5. Ngo LY, Kasahara S, Kumasaka DK, Knoblaugh SE, Jhingran A, Hohl TM. 2014. Inflammatory Monocytes Mediate Early and Organ-Specific Innate Defense During Systemic Candidiasis. The Journal of Infectious Diseases 209:109–119.
